# Associated Bacterial Coinfections in COVID-19-Positive Patients

**DOI:** 10.3390/medicina59101858

**Published:** 2023-10-19

**Authors:** Eugen Radu Boia, Alexandru Romulus Huț, Alexandra Roi, Ruxandra Elena Luca, Ioana Roxana Munteanu, Ciprian Ioan Roi, Mircea Riviș, Simina Boia, Adina Octavia Duse, Dan Dumitru Vulcănescu, Florin George Horhat

**Affiliations:** 1 Department of Ear, Nose and Throat, Faculty of Medicine, “Victor Babeș” University of Medicine and Pharmacy Timisoara, 2 Eftimie Murgu Sq., 300041 Timisoara, Romania; eugen.boia@umft.ro; 2 PhD Researcher, “Victor Babeș” University of Medicine and Pharmacy Timisoara, 2 Eftimie Murgu Sq., 300041 Timisoara, Romania; alexandru.hut@umft.ro; 3 Department of Oral Pathology, Faculty of Dental Medicine, Multidisciplinary Center for Research, Evaluation, Diagnosis and Therapies in Oral Medicine, “Victor Babeș” University of Medicine and Pharmacy Timisoara, 2 Eftimie Murgu Sq., 300041 Timisoara, Romania; alexandra.moga@umft.ro; 4 Department of Oral Rehabilitation and Dental Emergencies, Faculty of Dental Medicine, The Interdisciplinary Center for Dental Medical Research, Lasers and Innovative Technologies, “Victor Babeș” University of Medicine and Pharmacy Timisoara, 2 Eftimie Murgu Sq., 300041 Timisoara, Romania; luca.ruxandra@umft.ro (R.E.L.); muteanu.roxana@umft.ro (I.R.M.); 5 Department of Anesthesiology and Oral Surgery, Faculty of Dental Medicine, Multidisciplinary Center for Research, Evaluation, Diagnosis and Therapies in Oral Medicine, “Victor Babeș” University of Medicine and Pharmacy Timisoara, 2 Eftimie Murgu Sq., 300041 Timisoara, Romania; ciprian.roi@umft.ro (C.I.R.); mircea.rivis@umft.ro (M.R.); 6 Department of Periodontology, Faculty of Dental Medicine, Anton Sculean Research Center for Periodontal and Peri-Implant Diseases, “Victor Babeș” University of Medicine and Pharmacy Timisoara, 2 Eftimie Murgu Sq., 300041 Timisoara, Romania; 7 Department of Physical Medicine, Balneology and Rheumatology, Faculty of Medicine, Center for the Evaluation of Movement, Functionality and Disability, “Victor Babeș” University of Medicine and Pharmacy Timisoara, 2 Eftimie Murgu Sq., 300041 Timisoara, Romania; duse.adina@umft.ro; 8 Department of Microbiology, Faculty of Medicine, Multidiciplinary Research Center on Antimicrobial Resistance (MULTI-REZ), “Victor Babeș” University of Medicine and Pharmacy Timișoara, 2 Eftimie Murgu Sq., 300041 Timisoara, Romania; dan.vulcanescu@umft.ro (D.D.V.); horhat.florin@umft.ro (F.G.H.)

**Keywords:** COVID-19, cervical pathology, oral pathogens

## Abstract

*Background and Objectives*: The aim of this study was to identify specific rhino- and oropharyngeal microbiological pathogens as well as associated comorbidities that favor SARS-CoV-2 infection and corelate them. *Materials and Methods*: This prospective clinical study enrolled 61 patients (28 COVID-19-positive and 33 controls) who were tested for other comorbidities and co-existence of associated oral pathogenic microbiota. *Results*: A total of 247 bacterial isolates were identified in the bacterial cultures in both groups. Viral hepatitis type A was more prevalent in the COVID-19-positive group (*p* = 0.026), as was the presence of oral candidiasis (*p* = 0.006). In the control group, a moderate direct relationship was observed between the *Beta hemolytic streptococcus* group G and dermatitis, and strong direct relationships were observed between the *Beta hemolytic streptococcus* group G and external otitis, *Streptococcus pyogenes* and dental alveolitis, and *Streptococcus pyogenes* and chronic lymphocytic leukemia. In the test group, strong direct relationships were observed between *Hemophilus influenzae* and pulmonary thromboembolism; *Staphylococcus aureus* and autoimmune thyroiditis; post-viral immunosuppression, chronic coronary syndrome, and hypernatremia; *Beta hemolytic streptococcus* group C and rheumatoid polyneuropathy; *Beta hemolytic streptococcus* group G and hyperkalemia, hypothyroidism, secondary anemia, and splenomegaly; and active oral candidiasis and SARS-CoV-2 viral pneumonia. The following relationships were strong, but inverse: *Beta hemolytic streptococcus* group G and acute respiratory failure, and active oral candidiasis and SARS-CoV-2 viral bronchopneumonia. *Conclusions*: Briefly, COVID-19-positive patients have the predisposition to build up associated comorbidities and coinfections, which can be the expression of the immune burden that this virus generates to the host.

## 1. Introduction

Coronavirus disease 2019 (COVID-19) has spread exponentially across the world. The most commonly typical manifestations of COVID-19 include fever, dry cough, headache, fatigue, sore throat, myalgia, abnormality in olfactory/gustatory senses, and gastrointestinal issues; less commonly, skin lesions and sputum production can occur [1,2].

The human coronavirus SARS-CoV-2, causing COVID-19 disease, is a respiratory virus that uses the oropharynx as the primary site of replication [3]. The oral cavity, being the entry point to the body, may be an active site of infection and an important reservoir of SARS-CoV-2, playing a critical role in its pathogenesis; however, it is unclear whether SARS-CoV-2 infection can alter the local homeostasis of the resident microbiota, actively cause dysbiosis, or influence cross-body site interactions [4,5]. Considering that the oral surfaces are colonized by a diverse microbial community (bacteria, viruses, and fungi), it is likely that viruses have interactions with the host microbiota and can also influence SARS-CoV-2 infection [4]. Periodontal-associated cytokines might drive the alteration of the respiratory epithelium via the aspiration of oral pathogens into respiratory organs to promote the adhesion of the virus; therefore, the oral microbiome might impact lung infection and microbial colonization by SARS-CoV-2 [4,6,7,8].

Bacterial coinfections are not uncommon with respiratory viral pathogens, and although they are rare upon patient admission, they are frequent among patients requiring prolonged hospitalization for COVID-19, particularly those who require mechanical ventilation in an ICU and can add to significant mortality and morbidity [9].

Although the disease results in mild symptoms in most cases, it progresses to severe pneumonia and multi-organ failure, leading to mortality in some cases depending on patient age and the presence of comorbidities [10,11,12,13,14,15]. Independent risk factors, such as elevated procalcitonin and C-reactive protein levels, lymphopenia, leukocytosis, and acute kidney injury requiring dialysis, were identified as down-graders in SARS-CoV-2 viral infection evolution.

Additionally, more recent studies have reported clinical orofacial manifestations in COVID-19-positive patients, including oral ulcerative lesions, vesiculobullous lesions, and acute sialadenitis [1]. Due to resource constraints, overburdened healthcare systems, and current evidence suggesting lower rates of coinfections, it is plausible that patients infected with COVID-19 are simply not being evaluated for coinfections. Thus, the true coinfection rate may be higher than what is currently suggested by the available literature [16].

The aim of this study was to identify specific rhino- and oropharyngeal microbiological pathogens as well as associated comorbidities that favor SARS-CoV-2 infection and corelate them.

## 2. Materials and Methods

### 2.1. Study Population

Between October 2021 and November 2021, this descriptive and analytical trial enrolled 61 subjects (33 males and 28 females) who were outpatients at the Clinical Hospital of Infectious Diseases and Pneumophysiology “Dr. Victor Babeș Timișoara” and at the ENT Department of the Faculty of Medicine of the “Victor Babeş” University of Medicine and Pharmacy in Timișoara. The study protocol was approved by the Research Ethics Committee of the “Victor Babeş” University of Medicine and Pharmacy (approval no. 77/16.11.2021).

The study was conducted over a period of one and a half months (1 October 2021–12 November 2021) in accordance with principles outlined in the Declaration of Helsinki on experimentation involving human subjects. All subjects were informed about the nature and purpose of the study, and each subject signed an informed consent document giving their permission for the sampling of biological material. The study population consisted of males and females (mean age, 62.39 ± 15.61 years; range, 20 to 90 years).

### 2.2. Microbiological Sampling

Hospitalized COVID-19-positive patients (*n* = 28) and matched healthy controls (*n* = 33) underwent rhino-pharyngeal, oropharyngeal for diagnosis of SARS-CoV-2, and oral swabs specimens touching tongue, palate, and cheeks, and then they were additionally collected for oral microbiota identification. The history of hepatitis A was collected upon admission in the hospital evaluating each patient’s medical history. The clinical standard admission protocol in the hospital requires complete blood counts and antibiogram.

Identification of SARS-CoV-2 was carried out using real-time polymerase chain reaction (real-time PCR) analysis performed on a BIO-RAD CFX96 Real-Time System C1000 Touch Thermal Cycler Device. For bacteria, isolation was conducted on Columbia 5% sheep blood agar (Sanimed, Bucharest, Romania). Identification of all isolates was performed according to the morphological characters of colonies and their biochemical tests obtained using the automated VITEK 2 system (bioMérieux, Inc., Marcy-l’Étoile, France).

### 2.3. Statistical Analysis

Minimum sample size was calculated using G*Power (v. 3.1.9.6, Kiel University, Kiel, Germany) for the following parameters: power = 80%; effect size 0.5. The minimum calculated sample size was 52. The number of enrolled patients was larger than the minimum required sample size (*n* = 61).

Normality was assessed using the Shapiro–Wilk test, which was rejected for all parameters. As such, non-parametric tests were chosen. Results are presented as median values and interquartile ranges (IQRs). For comparison of these data, the Mann–Whitney U test was used. Associative testing was performed using Spearman’s rank correlation, the interpretation of the results being observable in Table 1.

Categorical data are presented as number and frequency. Contingency tables were created and the Chi^2^ test was used to check for associations between table rows and columns. For cases of variable values below 5, Fisher’s exact test was used instead. All *p* values < 0.05 were regarded as statistically significant. Statistical analysis was performed using MedCalc^®^ Statistical Software version 20.216 (MedCalc Software Ltd., Ostend, Belgium).

## 3. Results

There were 33 (54.10%) COVID-19-negative and 28 (45.90%) -positive patients. There were 33 (54.10%) males and 28 (45.90%) females in total. Regarding age, the median value was 53 and the IQR was 34 (36.25–70.25). Also, patients with COVID-19 were older (median = 61.5, IQR = 25.5, 50–75.5) than COVID-19-negative patients (median = 45, IQR = 36, 27–63), a difference which proved statistically significant (*p* < 0.001). A total of 247 bacterial isolates were identified in the bacterial cultures in both groups.

When checking for associations, using the Chi^2^ test, the following relationships could be observed: COVID-19 and sex (*p* = 0.049), with more males being in the positive group; COVID-19 patients and history of viral hepatitis A (*p* = 0.03926), with history of viral hepatitis A being more prevalent in the positive group; and COVID-19 patients and active oral candidiasis (*p* = 0.0076), with more patients with active candidiasis being in the positive group. This is detailed in Table 2.

### 3.1. COVID-19-Negative Patients

Of the total 33 healthy controls, there were 14 (42.42%) males and 19 (67.86%) females. For this group, the Spearman’s rank correlation test was used, and the statistically significant results are displayed in Table 3. As such, a moderate direct relationship was observed between *Beta hemolytic streptococcus* group G and dermatitis, and strong direct relationships were observed between *Beta hemolytic streptococcus* group G and external otitis, *Streptococcus pyogenes* and dental alveolitis, and *Streptococcus pyogenes* and chronic lymphocytic leukemia.

### 3.2. COVID-19-Positive Patients

Of the total 28 COVID-19-positive patients, there were 19 (67.86%) males and 9 (32.14%) females. For this group, the Spearman’s rank correlation test was used, and the statistically significant results are displayed in Table 4. Moderate direct relationships were observed between Beta hemolytic streptococcus group C and hepatocytolysis and *Beta hemolytic streptococcus* group G and hyperkalemia, while a moderate inverse relationship was observed between *Beta hemolytic streptococcus* group G and asthenic syndrome.

Strong direct relationships were observed between Hemophilus influenzae and pulmonary thromboembolism, *Staphylococcus aureus* and the female sex, *Staphylococcus aureus* and autoimmune thyroiditis, *Staphylococcus aureus* and post-viral immunosuppression, *Staphylococcus aureus* and chronic coronary syndrome, *Staphylococcus aureus* and hypernatremia, *Beta hemolytic streptococcus* group C and rheumatoid polyneuropathy, *Beta hemolytic streptococcus* group G and hyperkalemia, *Beta hemolytic streptococcus* group G and hypothyroidism, *Beta hemolytic streptococcus* group G and secondary anemia, *Beta hemolytic streptococcus* group G and splenomegaly, and active oral candidiasis and SARS-CoV-2 viral pneumonia. The following relationships were strong, but inverse: *Beta hemolytic streptococcus* group G and acute respiratory failure, and active oral candidiasis and SARS-CoV-2 viral bronchopneumonia.

## 4. Discussion

Several recent studies highlighted the potential role of the oral microbiome in triggering different bacteria of viral infection; however, the particularity of this mechanism is vaguely defined. The novel concept of “the keystone pathogen” is considered to be involved in the complex process of dysbiotic diseases by coordinating the pathogenic microbiota trough the saprophytic one [17].

Early studies suggested that men and women were equally susceptible to COVID-19 infection; however, men are at higher risk for severe symptoms and death, a fact that is also observed in our research [18]. This may be caused by the inherited differences in the systemic immune responses of the innate and adaptive immune system, rendering them more susceptible to an unfavorable response to infection [18,19,20].

In the present study, positive correlations were found between acute COVID-19 infection and hepatitis A virus presence, a parallel also demonstrated by other authors [21] and also with hepatitis B [22] and E [23].

The presence of Candida albicans in the oral cavity can be totally benign, being part of the usual commensal flora; however, its pathogenicity is validated as opportunistic when immune status is compromised, like in the case of COVID-19 infection [24]. The incidence of oral candidiasis is frequent in hospitalized patients with COVID-19 that undergo broad-spectrum antibiotic or prolonged corticosteroid treatment [25,26,27,28]. Our results also confirmed that there is a solid incidence of oral candidiasis in the positive group (*p* = 0.006).

Infection with Candida albicans in COVID-19 patients was also strongly correlated positively with pneumonia (R = 0.561, *p* = 0.002) but inversely with bronchopneumonia (R = −0.535, *p* = 0.003). This is important, as other studies have pointed out, to the risk that these patients have in regard to possible system infections due to fungal agents [29]. Also, the relationship between SARS-CoV-2 viral pneumonia and oral candidiasis was observed by other authors as well [24].

Although, in our study, the Chi^2^ test could not establish a relationship between bacterial agents and COVID-19 infection, which was observed by other authors [30,31,32,33], several relationships could be observed between the studied pathogens and several comorbidities, especially in the COVID-19-positive group.

Gram-positive pathogens like Streptococcus can induce various infections in humans. There are many different types of streptococci, their classification being made into groups based on their cell wall antigens. Infections with group C and G streptococci are far less common and understood than those with group A and B, yet they can cause pharyngitis [34], skin infections [35,36,37], and bacteremia [38]. Even if there are no specific data supporting that streptococcus beta hemolytic group G clearly causes external otitis, our findings showed a strong relationship between these two (*p* < 0.001).

Another member of the Streptococcus family investigated in our research is *Streptococcus pyogenes*, of which its presence was strongly correlated with dental alveolitis, a fact confirmed by many other authors [39,40,41]. Nevertheless, in immunosuppressant diseases like chronic lymphocytic leukemia, patients are prone to infections because of both humoral immunodepression, innate to the hematologic disease, and the immunosuppression related to the administrated treatment [42]; hence, the activity of co-infections is exacerbated.

Although prior studies identified several pathways contributing to pulmonary thromboembolism, it is unknown whether this is COVID-19-specific or also occurs in acute respiratory distress syndrome patients with another infection like Hemophilus influenzae [43]. Nevertheless, COVID-19 patients are at an increased risk of pulmonary thromboembolism development [44].

COVID-19-positive women are more likely to develop Staphylococcus infections, as proved by a moderate direct relationship (R = 0.503, *p* = 0.006) in the Spearman’s test. However, there are no data regarding the prevalence of *S. aureus* in women specifically [45,46]. A multicenter, retrospective cohort study showed that *Staphylococcus aureus* bacteremia causes significant morbidity and mortality in patients with pre-existing immunosuppression, an aspect confirmed by our study as well [47]. *Staphylococcus aureus* is not typically associated with chronic coronary syndrome, which is a pathological process characterized by atherosclerotic plaque accumulation in the epicardial arteries; however, some studies have shown that there may be an association between this latter cardiac pathology and bacterial infections such as Chlamydia pneumoniae and Helicobacter pylori [48,49,50]. We found a strong correlation between chronic coronary syndrome and the presence of *Staphylococcus aureus* in the COVID-19-positive group.

Hypernatremia is a condition that generates an excess of sodium in the bloodstream and can be caused by many factors including dehydration, kidney disease, and certain medications like diuretics [51] and corticosteroids [52]. Even though there is not any direct evidence in the literature that there is a link between *Staphylococcus aureus* and hypernatremia, it is possible that the infection with this pathogen could lead to dehydration, which might then lead to hypernatremia.

No direct link in the literature was found for *Beta hemolytic streptococcus* group C and hepatocytolysis; however, it is possible that this pathogen can cause liver damage in some cases [53]. Interestingly, although a very important complication in COVID-19-positive patients is represented by acute respiratory failure, it was inversely and strongly correlated (R = −0.694, *p* < 0.001) with the oral presence of *Beta hemolytic streptococcus* group G, which can cause respiratory infections in rare cases. The same can be said about the relationship between the presence of *Beta hemolytic streptococcus* group G in oral samples and the presence of asthenic syndrome; however, this relationship was considered to be moderate (R = −0.403. *p* = 0.034). No such relationships have been previously described; however, a shift in microbiota due to the severity of COVID-19 has been observed in the literature and may be the mechanism at play in this regard [54,55]. Also, a study by Islam et al. pointed to a correlation of high association of streptococcus spp. with secondary bacterial lung infection, breathing difficulty, and sore throat [56].

## 5. Limitations

Firstly, although the minimum sample size was achieved and overcome, compared to other research, our sample size was moderate. A larger sample size would provide more in-depth information. Secondly, although we used the Vitek 2 automated system for the identification of microbial agents, identification using PCR or another molecular biology method might have provided more sensitive in this regard.

## 6. Conclusions

We can conclude that COVID-19-positive patients have a tendency to develop more associated comorbidities and coinfections. This might be the expression of the immune burden that this virus provokes to the host, which can be amplified by the effects of the medication for the treatment of this systemic viral infection.

## Figures and Tables

**Table 1:** Interpretation of the R values.

Spearman R	Correlation
>0.75	Very strong
0.50–0.74	Strong
0.25–0.49	Moderate
0.01–0.25	Weak

**Table 2:** The results of the contingency table testing.

Parameter	COVID-19-Negative	COVID-19-Positive	Chi^2^ Test (*p*)
Male	14 (42.42%)	19 (67.86%)	0.049
Female	19 (57.58%)	9 (32.14%)	
Urban	19 (57.58%)	16 (57.14%)	0.973
Rural	14 (42.42%)	12 (42.86%)	
**Parameter**	**COVID-19-negative**	**COVID-19-positive**	**Fisher’s exact test (*p*)**
*S. Aureus* positive	2 (6.06%)	3 (10.71%)	0.653
*S. Aureus* negative	31 (93.94%)	25 (89.29%)	
*Str. Pyogenes* positive	3 (9.09%)	0 (0.00%)	0.243
*Str. Pyogenes* negative	30 (90.91%)	28 (100.00%)	
*Str. Beta-hemolytic group G* positive	4 (12.12%)	2 (7.14%)	0.678
*Str. Beta-hemolytic group G* negative	29 (87.88%)	26 (92.86%)	
*Str. Beta-hemolytic group C* positive	5 (15.15%)	3 (10.71%)	0.715
*Str. Beta-hemolytic group C* negative	28 (84.85%)	25 (89.29%)	
*Hemophilus influenzae* positive	0 (0.00%)	1 (3.57%)	0.459
*Hemophilus influenzae* negative	33 (100.00%)	27 (96.43%)	
History of A viral hepatitis positive	0 (0.00%)	4 (14.29%)	0.039
History of A viral hepatitis negative	33 (100.00%)	24 (85.71%)	
Active oral candidiasis positive	0 (0.00%)	6 (21.43%)	0.007
Active oral candidiasis negative	33 (100.00%)	22 (78.57%)	

**Table 3:** The results of the Spearman’s rank correlation test for healthy controls.

Pathogen	Comorbidities	R	Lower CI	Higher CI	*p*
*Beta hemolytic streptococcus* group G	Dermatitis	0.476	0.159	0.704	0.005
	External otitis	0.558	0.260	0.759	<0.001
*Streptococcus pyogenes*	Dental alveolitis	0.559	0.267	0.757	<0.001
	Chronic lymphocytic leukemia	0.559	0.267	0.757	<0.001

**Table 4:** The results of the Spearman’s rank correlation test for the positive group.

Pathogen	Comorbidities	R	Lower CI	Higher CI	*p*
*Hemophilus influenzae*	Pulmonary thromboembolism	0.694	0.433	0.848	<0.001
*Staphylococcus aureus*	Women	0.503	0.160	0.738	0.006
	Autoimmune thyroiditis	0.556	0.230	0.769	0.002
	Post-viral immunosuppression	0.556	0.230	0.769	0.002
	Chronic coronary syndrome	0.556	0.230	0.769	0.002
	Hypernatremia	0.556	0.230	0.769	0.002
*Beta hemolytic streptococcus* group C	Hepatocytolysis	0.441	0.082	0.699	0.019
	Rheumatoid polyneuropathy	0.556	0.230	0.769	0.002
*Beta hemolytic streptococcus* group G	Acute respiratory failure	−0.694	−0.848	−0.433	<0.001
	Asthenic Syndrome	−0.403	−0.675	−0.035	0.034
	Hyperkalemia	0.462	0.107	0.712	0.013
	Hypothyroidism	0.694	0.433	0.848	<0.001
	Secondary anemia	0.694	0.433	0.848	<0.001
	Splenomegaly	0.694	0.433	0.848	<0.001
Active oral candidiasis	SARS-CoV-2 viral pneumonia	0.561	0.238	0.772	0.002
	SARS-CoV-2 viral bronchopneumonia	−0.535	−0.757	−0.203	0.003

## Data Availability

The datasets used/analyzed during the current study are available from the corresponding author on reasonable request.
